# Identification of NET formation and the renoprotective effect of degraded NETs in lupus nephritis

**DOI:** 10.1152/ajprenal.00122.2024

**Published:** 2024-08-29

**Authors:** Yong Jin, Yutong Wang, Xu Ma, Hongbin Li, Manling Zhang

**Affiliations:** ^1^Department of Rheumatology and Immunology, The Affiliated Hospital of Inner Mongolia Medical University, Hohhot, China; ^2^Inner Mongolia Key Laboratory for Pathogenesis and Diagnosis of Rheumatic and Autoimmune Diseases, The Affiliated Hospital of Inner Mongolia Medical University, Hohhot, China

**Keywords:** core genes, lupus nephritis, neutrophil activation pathway, neutrophil extracellular traps, PBMCs and renal tissues

## Abstract

To explore molecular biomarkers associated with the pathophysiology and therapy of lupus nephritis (LN), we conducted a joint analysis of transcriptomic data from 40 peripheral blood mononuclear cells (PBMCs) (GSE81622) and 21 kidney samples (GSE112943) from the Gene Expression Omnibus database using bioinformatics. A total of 976 and 2,427 differentially expressed genes (DEGs) were identified in PBMCs and renal tissues. Seven and two functional modules closely related to LN were identified. Further enrichment analysis revealed that the neutrophil activation pathway was highly active in both PBMCs and the kidney. Subsequently, 16 core genes closely associated with LN were verified by protein-protein interaction screening and quantitative PCR. In vitro cell models and MRL/lpr mouse models confirmed that the abnormal expression of these core genes was closely linked to neutrophil extracellular traps (NETs) generated by neutrophil activation, while degradation of NETs led to downregulation of core gene expression, thereby improving pathological symptoms of LN. Therefore, identification of patients with systemic lupus erythematosus exhibiting abnormal expression patterns for these core genes may serve as a useful indicator for kidney involvement. In addition, targeting neutrophils to modulate their activation levels and inhibit aberrant expression of these genes represents a potential therapeutic strategy for treating LN.

**NEW & NOTEWORTHY** The mechanisms by which immune cells cause kidney injury in lupus nephritis are poorly understood. We integrated and analyzed the transcriptomic features of PBMCs and renal tissues from the GEO database to identify key molecular markers associated with neutrophil activation. We confirmed that neutrophil extracellular traps (NETs) formed by neutrophil activation promoted the upregulation of key genes in cell and animal models. Targeted degradation of NETs significantly ameliorated kidney injury in MRL/lpr mice.

## INTRODUCTION

Lupus nephritis (LN) is one of the most prevalent complications of systemic lupus erythematosus (SLE). After development into end-stage renal disease, it has a high mortality rate, rendering it a common clinical life-threatening critical illness (1, 2). LN is primarily caused by the formation and deposition of immune complexes in the kidneys as well as aberrant expression of immune cells and immune proteins, which induce inflammatory damage of glomeruli, renal tubules, and the renal interstitium and then lead to renal insufficiency (3, 4). Due to the complexity and heterogeneity of LN, this poses challenges for determining its pathogenesis and developing effective treatments.

Transcriptomic analysis is a potent tool for the discovery of new targets and exploration of new therapeutic approaches in various diseases, including LN (5). In LN, several new LN targets and mechanisms have been identified by transcriptomics approaches (6–8). However, the transcriptomics method has certain limitations across different studies, such as the use of a single type of sample and small sample size, differences in gene screening, large errors, poor stability, and other factors. Bioinformatics conjoint analysis can enhance the statistical power of the study by integrating multiple transcriptomic methods and data to obtain a larger number of samples and more differentially expressed genes (DEGs) (9, 10) Bioinformatics conjoint analysis has been used in many studies to analyze existing transcriptomics data and identify some crucial molecular markers. Yao et al. (5) integrated glomerular and renal tubular interstitial transcriptomics data from patients with LN and found that the expression of some key genes influences interaction of signaling pathways between glomerular and tubular interstitial. Cao et al. (11) found that the expression level of OAS family-related genes in glomeruli was closely associated with disease progression in LN. Based on bioinformatics conjoint analysis, it was found that the infiltration of immune cells such as monocytes in the glomerulus also played a significant role in LN pathogenesis (12). All the above studies used renal biopsy samples for transcriptome sequencing and analysis, while noninvasive samples capable of reflecting pathological processes of the disease are widely accepted by clinical and basic research.

Many studies have used transcriptomic approaches to compare the gene expression in peripheral blood mononuclear cells (PBMCs) from patients with SLE against those from healthy individuals or patients with other autoimmune diseases. This provides great help in finding diagnostic and prognostic biomarkers for autoimmune diseases (13, 14). Researchers have identified MX1, GPR84, and E2F2 as potential LN biomarkers through transcriptome sequencing analysis of PBMC samples from patients, which are highly expressed in LN and lowly expressed in patients with non-LN SLE with high specificity (15). Recently, bioinformatics conjoint analysis has revealed that five type I interferon (IFN)-related genes IFI44, IFI44L, IFIT1, MX1, and USP18 in PBMCs play a crucial role as methylated epigenetic drivers of SLE and LN. This finding further supports the significance of the type I IFN pathway in the pathogenesis of SLE (16). These studies suggested that PBMCs as a type of immune cell may reflect the pathological process of LN to some extent through its gene expression. However, renal inflammatory injury is mediated by a variety of immune cells, which are either derived from kidney-resident immune cells or from peripheral blood-infiltrating immune cells (17–19), and there is a close correlation between them. Therefore, the integration of gene expression data from peripheral blood immune cells and kidney tissue will be able to reflect the occurrence and development of the disease more comprehensively. To our knowledge, few studies have used bioinformatics tools to jointly analyze kidney tissue and PBMC transcriptomic datasets from patients with LN to discover new potential biomarkers and understand the pathogenesis of LN. Therefore, it is attractive and highly meaningful to do so.

In response to strong signals, neutrophils activate and release extracellular DNA structures decorated with various protein substances, called neutrophilic extracellular traps (NETs) (20). Initially, NET formation was thought to be a unique mechanism of host defense and pathogen destruction (21, 22). However, further research found that the formation of NETs in the kidneys was a major driver of the self-amplifying cycle of tissue necrosis and inflammation (23). Studies have demonstrated that neutrophils can also infiltrate the kidneys of active LN and induce a resident inflammatory response, primarily through the formation of NETs (7, 24). NETs were also present in the kidneys of patients with LN that may serve as a source of antigenic nucleosomes and promote immune complex formation (25). Mechanism studies have shown that NETs can further trigger autoimmune responses in SLE by exposing histone-DNA complexes that promote the synthesis of type I IFNs by plasmacytoid dendritic cells by activating the Toll-like receptor (TLR) in endosomes (26, 27). Recent studies on the LN gene regulatory network showed that the expression of type I IFN-stimulating genes (ISGs) was also significantly activated in neutrophils (28). However, a comprehensive and integrated exploration of the gene regulation associated with NETs in LN is still lacking.

In this study, we integrated and analyzed the transcriptomic features of 40 PBMC samples and 21 renal tissue samples in the Gene Expression Omnibus (GEO) database to identify key molecular markers and investigate the pathogenesis of LN. First, we used R language software for extracting transcriptome profiles, performing cross-platform normalization, and identifying DEGs. Subsequently, we used CIBERSORT and WGCNA algorithms to analyze immune cell infiltration and coexpression of disease-related genes. Through Gene Ontology (GO), Kyoto Encyclopedia of Genes and Genomes (KEGG) pathway, and protein-protein interaction (PPI) network analysis, we identified the key upregulated genes in both PBMCs and kidney tissues. Notably, most of these genes were found to be associated with neutrophil activation. Furthermore, NETs formed by neutrophil activation promoted the upregulation of core genes in samples from patients with LN, PBMCs, and lupus mouse models. Finally, targeted degradation of NETs significantly reduced expression levels of core genes and ameliorated kidney injury in a mouse model of LN.

## MATERIALS AND METHODS

### Dataset Selection, Preprocessing, and Identification of DEGs

To obtain the transcriptome data related to human LN, we downloaded gene expression spectrum matrix files (GSE81622 and GSE112943) from the GEO database (https://www.ncbi.nlm.nih.gov/geo/). Based on these datasets, a total of 15 PBMC samples from patients with LN, 25 PBMC samples from normal controls, 14 kidney samples from patients with LN, and seven kidney samples from normal controls were included in this study (Supplemental Table S1). Perl language commands were used to convert gene probe IDs in matrix files into genome symbols in platform files, resulting in matrix files containing international standard gene names. Subsequently, we used the limma R package (http://www.bioconductor.org/) with the normalize Between Arrays functions for normalization of each dataset followed by log_2_ |fold change (FC)| transformation of all gene expression data (Supplemental Tables S2-1 and S2-2).

The limma R software package was used to screen DEGs in each dataset. A gene was considered as a DEG when correcting *P* value < 0.05 and log_2_ |FC|>2. The list of upregulated and downregulated genes was saved as an Excel file, and the DEGs in each dataset were sorted by logFC and saved in an Excel file for subsequent relevant analysis (Supplemental Tables S3-1 and S3-2).

### Weighted Gene Coexpression Network Analysis

To more accurately investigate the functionality of DEGs, we identified coexpression modules in PBMC and kidney samples using weighted gene coexpression network analysis (WGCNA), an algorithm that specifically screens genes associated with clinical features to obtain highly biologically significant coexpression modules (29). We selected DEGs based on log_2_ |FC| > 2 and *P* < 0.05 criteria for further analysis using the WGCNA R package.

First, the “pickSoftThreshold” function was used to determine the appropriate soft powers (β). Then, adjacent coefficients (*aij*) were calculated using the following equation: *aij* = |*Sij*| β, where *Sij* represents the Pearson correlation coefficient of genes *i* and *j*. Subsequently, based on adjacency coefficient *aij*, a topological overlap matrix (TOM) and corresponding dissimilarity (1-TOM) were computed. Then, the coexpressed genes were divided into different modules using a hierarchical clustering dendrogram constructed based on the 1-TOM matrix. Finally, module eigen (ME) genes representing the expression pattern of each module were calculated, and Pearson correlation analysis was performed with clinical features to obtain modules significantly associated with LN.

In this study, the soft power was defined as 12 by WGCNA analysis of PBMCs and 14 by WGCNA analysis of the kidney. Other parameters are as follows: min module size = 30, network type = “unsigned,” deep split = 3, and merge cut height = 0.35.

### Functional Enrichment Analysis

GO analysis was used to describe the attributes of genes and gene products, encompassing biological processes (BPs), molecular functions (MFs), and cellular components (CCs). KEGG pathway enrichment analysis was used to obtain gene-level pathways. For the coexpressed modules obtained from WGCNA, we focused on DEGs that were significantly positively correlated with LN modules and performed GO and KEGG analysis of DEGs using the ggplot R package. We focused on the GO analysis results related to BP and KEGG pathways, and *P* values represented the significance of GO terms and pathways; the smaller the *P* value, the higher the significance.

### Immune Cell Infiltration Analysis

The CIBERSORT algorithm is an analytic tool used to estimate the proportion of various types of immune cells in complex tissues, such as large solid tumors (30), and this algorithm has been successfully applied to estimate the proportion of blood immune cell subsets in patients with SLE (31). In our study, we used the leukocyte gene signature matrix (LM22) to distinguish between 22 human hematopoietic cell phenotypes for identifying the infiltration of immune cells (32). The normalized data were analyzed using the CIBERSORT R software package. The Wilcoxon rank-sum test was used to compare the proportion of immune cells in LN PBMCs and kidney tissue with healthy samples, and *P* < 0.05 was considered significant.

### PPI Network Construction and Core Gene Analysis

Because kidney tissue and PBMCs are closely related in the pathogenesis of LN, we wondered whether the DEGs shared by both the modules identified by WGCNA of kidney tissue and PBMC samples would influence each other. First, we extracted DEGs from the modules positively associated with LN identified by WGCNA and screened for overlapping genes between kidney tissue and PBMC samples. Subsequently, we constructed PPI networks of hub genes at the protein level using the Search Tool for the retrieval of interacting genes (STRING) database. Our focus was on exploring interactions among hub genes within these overlapping genes. To identify key genes, Cytoscape software was used to screen hub genes according to degree (33). The MCODE plug-in in Cytoscape software was used to analyze significant modules within the PPI network using the following default parameters: “Degree Cutoff = 2,” “Node Score Cutoff = 0.2,” “K-Core = 2,” and “Max.Depth = 100” (34). We selected DEGs as core genes that met the following three restrictions: *1*) DEGs with a large FC (top 100); *2*) the gene was located in a critical module; and *3*) nodes with high MCODE scores determined by Cytoscape software. In addition, real-time quantitative PCR was performed to measure mRNA expression levels of core genes, and the steps of the whole process described above are shown in Supplemental Fig. S1.

### RNA Extraction and qPCR Analysis

Whole kidney tissues of mice in the C57BL/6J group, MRL/lpr group, and DNase I-treated MRL/lpr group were lysed to extract total RNA from the samples. In addition, PBMCs from three patients with LN and three healthy controls were also used for RNA extraction using a Promega kit. Subsequently, the extracted RNA was reverse-transcribed into cDNA using a reverse transcription kit (Vazyme). qPCR analysis was performed using qPCR Master Mix (Vazyme). Gene quantitative expression was normalized to β-actin or GAPDH. The FC of expression compared with the normal group was calculated using the 2^-△△Ct^ method (where C_t_ is threshold cycle). The primer details can be found in Supplemental Table S4.

### Animals

Animal experiments in this study were conducted in accordance with the approved experimental protocol by the Medical Ethics Committee of the Affiliated Hospital of Inner Mongolia Medical University. MRL/lpr female mice (6–8 wk old) and C57BL/6J female mice (8 wk old) were obtained from SPF Biological (Beijing, China). A total of 20 MRL/lpr mice were used for this study. In addition, 15 C57BL/6J mice were included as a control group. MRL/lpr mice were randomly divided into model groups at 13 wk, 17 wk, and 21 wk as well as DNase I treatment groups. C57BL/6J mice were used as a normal control group (*n* = 5/group). MRL/lpr mice exhibited varying degrees of alopecia at 17 wk of age. In the DNase I treatment groups, bovine DNase (No. 11284932001, Roche) at a concentration of 0.5 mg/mL, 150 μg (0.3 mL), was given on each occasion; this corresponds to ∼7.5 mg/kg (35). Intraperitoneal injections of DNase I were administered once daily, starting from the 17th week until euthanasia at the 21st week. Mice in both MRL/lpr 21-wk model group and normal control group received daily intraperitoneal injections of 0.3-mL normal saline for four consecutive weeks. Kidney and serum samples from MRL/lpr or C57BL/6J mice were collected at different time points (13 wk, 17 wk, and 21 wk) during the experiment and harvested at a consistent time of day (i.e., 9:00–11:00 AM).

### Collection and Biochemical Analysis of Serum and Urine Samples

From the 13th week onward, urine samples were collected at different time points (13, 17, and 21 wk) and the concentration of urinary protein was determined using the Coomassie brilliant blue (CBB) method (C035-2-1, Nanjing Jiancheng Bioengineering Institute). Serum samples were obtained from the MRL/lpr model at 13, 17, and 21 wk. The serum creatinine concentration was measured using the creatinase-coupled sarcoline oxidase method (C011-2-1, Nanjing Jiancheng Bioengineering Institute), while serum urea concentration was determined using the urease method (C013-2-1, Nanjing Jiancheng Bioengineering Institute). Serum anti-double-stranded DNA antibody (anti-dsDNA) (Cat. No. 5120, ADI) and serum anti-nuclear antibody (ANA) (Cat. No. 5210, ADI) concentrations were measured using ELISAs. Specific protocols for measuring serum and urine indexes can be found in each assay kit’s instructions.

### Histopathological Analysis

Mouse kidney tissues were fixed in formaldehyde solution for 2 days, dehydrated, embedded, and sectioned (2 µm). The pathological changes were observed through hematoxylin and eosin (HE) staining, periodic acid-Schiff (PAS) staining, and Masson staining. Positive areas were measured in five randomly selected fields, and images were captured using a light microscope (Leica). The pathological changes of renal tissues were scored semiquantitatively scored as follows: glomerular mesangial cell proliferation score ranging from 0 to 4 (0, none; 1, mild; 2, mild to moderate; 3, medium; and 4, severe); glomerular PAS^+^ deposition score ranging from 0 to 4 (0, none; 1, mild; 2, mild to moderate; 3, medium; and 4, severe); tubular lesion score ranging from 0 to 4 (0, none; 1, <25%; 2, 25–50%; 3, 50–75%; and 4, >75%), and score of crescent lesions ranged from 0 to 4 (0, none; 1, <25%; 2, 25–50%; 3, 50–75%; and 4, >75%) (36). The area of collagen positivity was quantified using ImageJ (National Institutes of Health).

### Immunohistochemistry

After paraffin sections were deparaffinized, 2-μm sections were immersed in water. Antigen repair was performed with citrate solution for 10 min at high temperature. Sections were blocked using sheep serum (ZLI-9022, ZSGB-BIO) for 1 h and then incubated with the primary polyclonal anti-mouse IgG (1:200, GB23301, Servicebio) or rabbit anti-OAS2 antibody (1:200, No. 19279-1-AP, Proteintech) diluted in blocking solution for 24 h (4°C) before proceeding to incubation with the secondary antibody. After the final DAB staining, positive areas were quantified in five randomly selected fields, and images were captured using a Leica light microscope. ImageJ software (National Institutes of Health) was used to quantify the positive areas for IgG deposition, and a repetitive method was adopted for semiquantitative scoring.

### Immunofluorescence

Neutrophils were isolated from the blood of patients with LN and healthy controls using ficoll density gradient centrifugation and then counted and placed in 24-well plates at a density of 5 × 10^4^ cells/well. Following overnight incubation for adherence, cells were divided into the control group, phorbol-12-myristate-13-acetate (PMA) treatment group, and PMA (25 ng/mL, P1585, Sigma) + DNase I (1 mg/mL, No. 11284932001, Roche) treatment group (37, 38). Four hours later, after fixation with 4% paraformaldehyde for 10 min at room temperature, permeabilization with 1% Triton X-100 for 1 h, and blocking with 5% BSA for 1 h at room temperature, primary antibodies against myeloperoxidase (MPO) (1:200, AB_396309, BD PharMingen) and CitH3 (1:200, ab5103, Abcam) were diluted in blocking solution and incubated overnight at 4°C. Secondary antibodies were diluted in blocking solution and incubated at room temperature for 1 h before DAPI (Sigma-Aldrich, St. Louis, MO) staining. A fluorescence microscope was used to observe and photograph. Paraffin-embedded biopsy sections of kidney tissue from different groups of mice were rehydrated, antigen repaired, and incubated with rabbit anti-MPO (1:200, AB_396309, BD PharMingen), mouse anti-CITH3 (1:200, ab5103, Abcam), or rabbit anti-IFI44L (1:200, PA5-97853, Invitrogen) antibodies.

### Western Blot Analysis

Western blot analysis was performed as previously described (39). Whole mouse kidney tissue lysates were prepared in RIPA buffer (Beyotime Biotechnology, Shanghai, China). Antibodies against the following proteins were used: IFI44L (1:1,000, PA5-97853, Invitrogen), OAS2 (1:1,000, No. 19279-1-AP, Proteintech), and RNASE1 (1:1,000, No. 15892-1-AP, Proteintech); GAPDH (1:3,000, HRP-60004, Proteintech) was used as a loading control.

### Statistical Analysis

GraphPad Prism 6.0 was used for conducting statistical analysis. At least three biological replicates were performed for each experiment. The measured data are presented as means ± SD. One-way ANOVA followed by Dunnett’s test or Bonferroni correction was conducted when the data involved three or more groups. *P* values of <0.05 were considered statistically significant.

## RESULTS

### Identification of DEGs in Patients With LN

**Figure 1.**
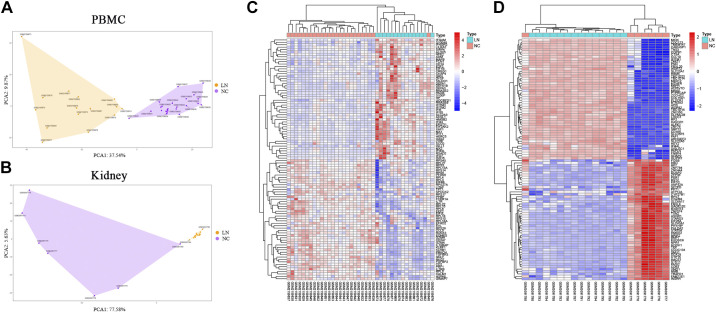
Data preprocessing and identification of DEGs in PBMC and kidney tissue. *A* and *B*: principal component analysis (PCA); sample clustering was performed between lupus nephritis (LN) groups and normal control (NC) groups. *C* and *D*: cluster heat map of the top 100 DEGs. Red indicates relative upregulation of gene expression; blue indicates relative downregulation of gene expression; white indicates no significant change in gene expression. PBMC: LN (*n* = 15) and NC (*n* = 25); kidney: LN (*n* = 14) and NC (*n* = 7). DEGs, differentially expressed genes; PBMC, peripheral blood mononuclear cell.

All microarray datasets were subjected to standardization and the results before and after standardization are presented in Supplemental Fig. S2, *A* and *B*. in addition, principal component analysis (PCA) demonstrated excellent biological repeatability and differences among the samples (Fig. 1, *A* and *B*). Based on the values of *P* < 0.05 and log_2_ |FC|>2, a total of 976 genes were identified as differentially expressed in the PBMC group of patients with LN, comprising 418 upregulated genes and 558 downregulated genes (Supplemental Fig. S2*C*). Similarly, a total of 2,427 genes were found to be differentially expressed in the kidney group of patients with LN with 1,577 upregulated genes and 850 downregulated genes (Supplemental Fig. S2*D*). The heatmap illustrates the top 50 upregulated and downregulated genes (Fig. 1, *C* and *D*).

### Coexpression Modules in Human PBMCs and the Kidney

**Figure 2.**
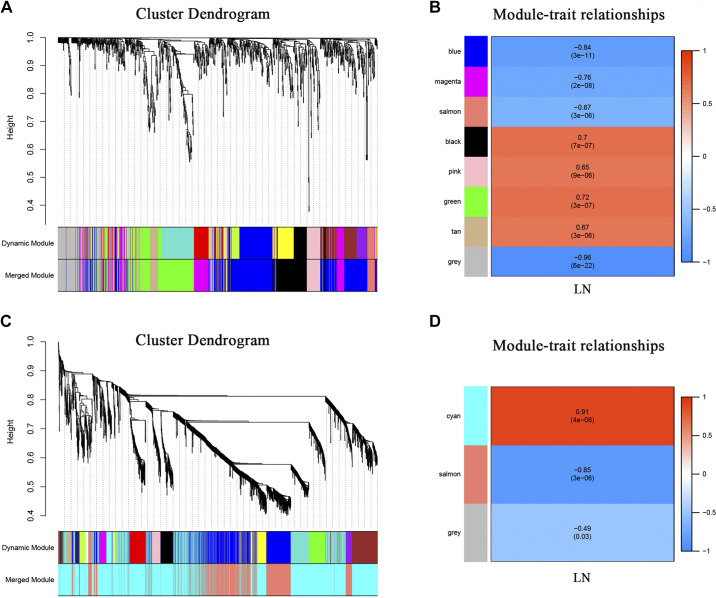
Weighted gene coexpression network analysis (WGCNA). *A*: cluster dendrogram of coexpression genes in PBMC. *B*: module-trait relationships in PBMC. *C*: cluster dendrogram of coexpression genes in kidney tissue. *D*: module-trait relationships in kidney tissue. Each cell contains the corresponding correlation and *P* value. Red represents positive correlation; blue represents negative correlation. LN, lupus nephritis; PBMC, peripheral blood mononuclear cell.

The WGCNA analysis revealed 976 genes in PBMCs and 2,427 genes in kidney tissue. Each module was assigned a color, and a total of seven modules were identified in PBMCs (excluding gray modules not assigned to any cluster). Heatmap of module-feature relationships was then generated to evaluate the association between each module and clinical features (LN). Figure 2, *A* and *B*, demonstrates that four modules (“black,” “pink,” “green,” and “brown”) exhibited positive correlations with LN, while three modules (“blue,” “magenta,” and “orange”) showed negative correlations with LN. Similarly, two modules were identified in the kidney; module “blue-green” displayed a positive correlation with LN, whereas module “orange” exhibited a negative correlation with LN (Fig. 2, *C* and *D*).

### GO and KEGG Pathway Analysis of Patients With LN

**Figure 3.**
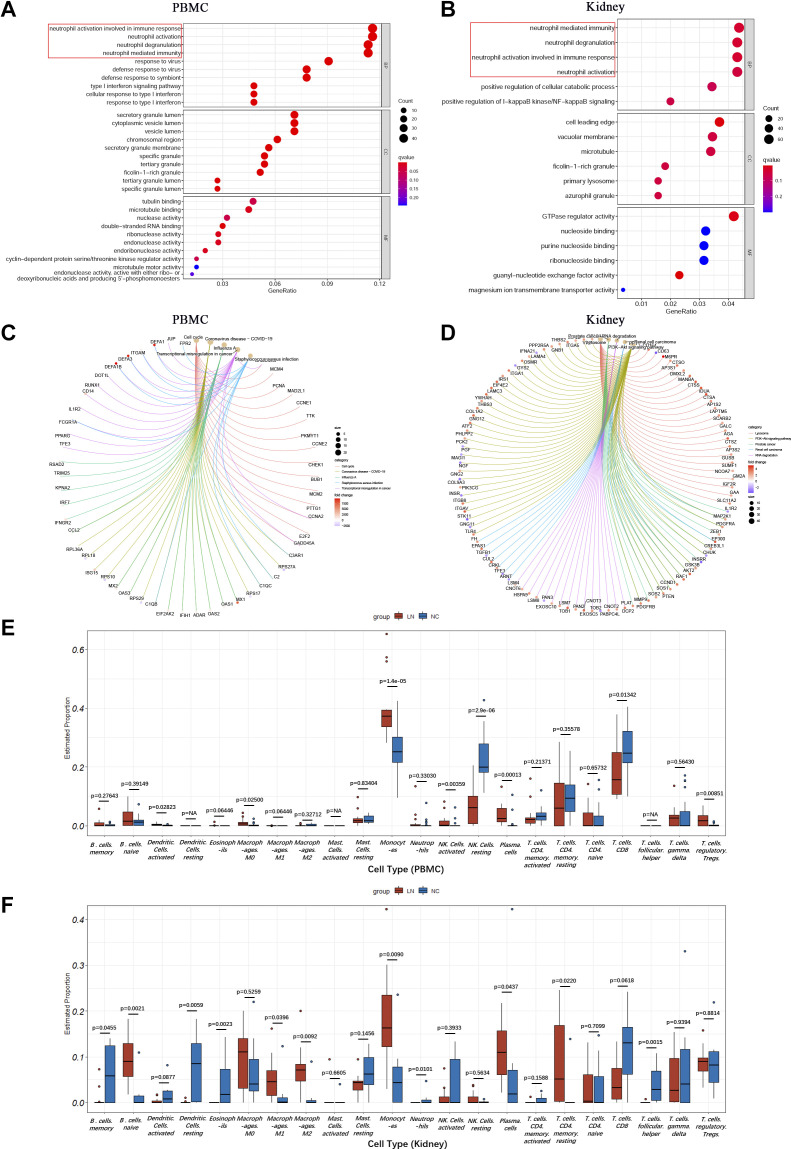
Functional enrichment analysis and immune cell infiltration analysis. *A* and *B*: enriched GO terms of DEGs in the modules positively related to LN in PBMC and kidney tissue. *C* and *D*: top 5 terms of KEGG analysis in the modules positively related to LN in PBMC and kidney tissue. *E* and *F*: proportion of immune cell infiltration in PBMC and kidney tissue. DEGs, differentially expressed genes; GO, Gene Ontology; KEGG, Kyoto Encyclopedia of Genes and Genomes; LN, lupus nephritis; NC, normal control; PBMC, peripheral blood mononuclear cell.

In PBMC, DEGs in the black, pink, green, and brown modules positively associated with LN were significantly enriched in immune responses, especially to neutrophil-mediated immune responses, such as “immune response involved in neutrophil activation,” “neutrophil activation,” and “neutrophil degranulation.” In addition, type I IFN-mediated immune responses to viral infection were also enriched, such as “response to virus,” “response to virus defense,” and “type I interferon signaling pathway.” KEGG pathway analysis revealed that abnormalities in signaling pathways associated with cell proliferation and induced by certain infectious diseases, such as cell cycle, coronavirus, influenza A, and *Staphylococcus aureus*. These signaling pathways were similar to the abnormality pathways during LN development (Fig. 3, *A* and *C*).

The DEGs in the blue-green module positively associated with LN in kidney tissue were also significantly enriched in neutrophil-mediated immune responses. Furthermore, cellular metabolism regulation and NF-κB inflammatory pathway were also enriched. Analysis of the KEGG pathway revealed that typical pathways associated with DEGs included lysosomal signaling, phosphatidylinositol 3-kinase-Akt signaling, renal cell proliferation, and so on (Fig. 3, *B* and *D*).

### Performance of Immune Cell Infiltration Analysis in Human PBMCs and the Kidney

As previously mentioned, the neutrophil-mediated immune response is very significant in LN. Considering that some related immune cells play a significant role, we used the CIBERSORT algorithm to estimate the proportion of various types of immune cells in PBMCs and the kidney and to explore their relationship with genes associated with neutrophil activation.

The results demonstrated a significant increase in the number of monocytes in PBMCs and kidneys of the LN group, as well as an elevated count of plasma cells, compared with the control group. Conversely, there was a reduction in the number of CD8 T cells. However, according to the CIBERSORT algorithm analysis, the number of neutrophils did not increase significantly in PBMCs and even showed a decrease due to the unmeasured value in the kidney (Fig. 3, *E* and *F*). In the kidney, the number of naive B cells, M1, and M2 macrophages increased, while the number of memory B cells, activated and resting dendritic cells, and T follicular helper cells decreased (Fig. 3*F*).

### Core Genes Identified From the Kidney and PBMCs of Patients With LN

**Figure 4.**
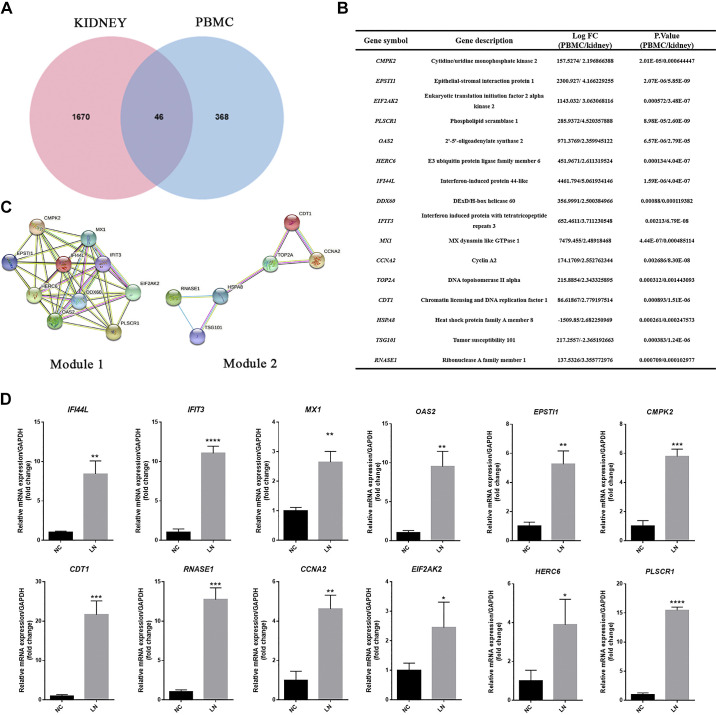
Core gene screening and identification. *A*: Venn diagram of DEGs in the positive correlation module of PBMC and kidney with LN. *B*: degree values of the 16 core genes. *C*: PPI network of *module 1* and *module 2*. Circles represent genes, lines represent interactions between gene-encoded proteins, and line colors represent evidence of interactions between proteins. *D*: mRNA expression of core genes in LN PBMC assessed by qRT-PCR. LN: *n* = 3 and NC: *n* = 3. Data are means ± SD for groups of three PBMC samples. *P < 0.05, **P < 0.01, ***P < 0.001, and ****P < 0.0001 vs. NC (*t* test). DEGs, differentially expressed genes; LN, lupus nephritis; NC, normal control; PBMC, peripheral blood mononuclear cell; PPI, protein-protein interaction.

As shown in the Venn diagram in Fig. 4*A*, there were a total of 46 DEGs in the modules identified by WGCNA of kidney and PBMC samples positively correlated with LN. The STRING online database was used to analyze these DEGs and construct the PPI network. We calculated the extent of each hub gene using the “Network Analysis” tool in Cytoscape 3.7.2. The 16 genes with the highest score were obtained, including CMPK2, EPSTI1, EIF2AK2, PLSCR1, OAS2, HERC6, IFI44L, DDX60, IFIT3, MX1, CCNA2, TOP2A, CDT1, HSPA8, TSG101, and RNASE1. Therefore, these genes were defined as “core” genes and the details of the core genes are shown in Fig. 4*B*.

In addition, two key functional modules were screened from the PPI network using MCODE analysis, as shown in Fig. 4*C*. Subsequently, we used qPCR to validate the expression of these core genes in PBMCs obtained from patients with LN and healthy controls. Remarkably, our findings revealed a significant upregulation of these genes in PBMCs derived from patients with LN compared with those from healthy controls (Fig. 4*D*), thereby indicating the reliability of the core genes we screened.

### MRL/lpr Mice Had Impaired Renal Function and Upregulated Expression of Related Core Genes

**Figure 5.**
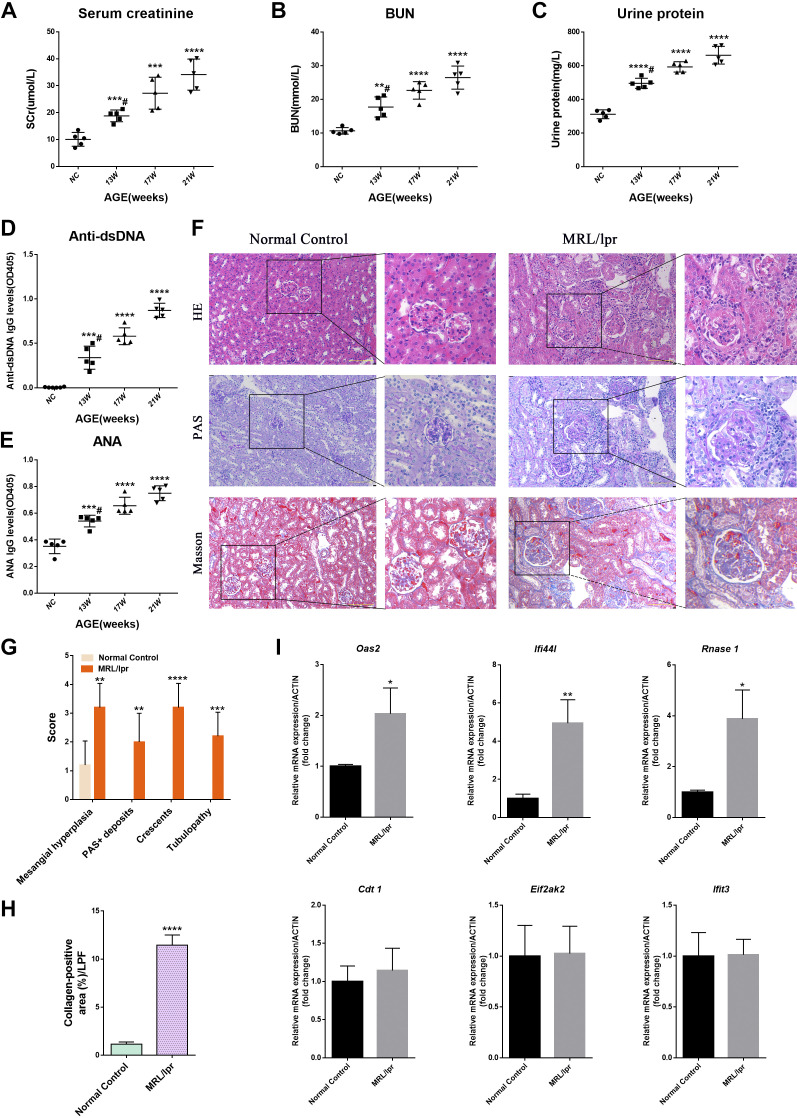
Core genes are upregulated in the kidney of MRL/lpr mice. *A–E*: serum creatinine (SCr) (*A*), blood urea nitrogen (BUN) (*B*), urine protein (*C*), anti-double-stranded DNA antibody (Anti-dsDNA) (*D*), and anti-nuclear antibody (ANA) (*E*) levels in NC and different weeks of MRL/lpr mice (*n* = 5). *F*: representative images of glomerular HE, PAS, and Masson staining in the NC group and 17 wk of MRL/lpr group. Bar = 100 μm. *G*: semiquantitative pathological score in the NC group and 17 wk of MRL/lpr group. *H*: quantification of the blue collagen-positive area (%) in both groups of mice. LPF, low-power field. *I*: mRNA expression of core genes in NC and 17 wk of MRL/lpr mouse kidneys assessed by qRT-PCR. Data are means ± SD for groups of five mice. *P < 0.05, **P < 0.01, ***P < 0.001, and ****P < 0.0001 vs. NC (*t* test). #*P* < 0.05 vs. 13 wk of MRL/lpr mice (Bonferroni correction; two comparisons were made). NC, normal control; 13 W, 13 weeks of MRL/lpr mice; 17 W, 17 weeks of MRL/lpr mice; 21 W, 21 weeks of MRL/lpr mice.

Compared with the normal control group, the serum creatinine, urea, urinary protein, anti-dsDNA, and ANA concentrations of lupus model MRL/lpr mice increased at the age of 13 wk, and the serum creatinine (Fig. 5*A*), serum urea (Fig. 5*B*), urinary protein (Fig. 5*C*), anti-dsDNA (Fig. 5*D*), and ANA (Fig. 5*E*) concentrations showed an upward trend with the increase of the week age of mice, suggesting that the renal function of lupus model mice was impaired. HE and PAS staining showed glomerular immune complex deposition, renal interstitial inflammatory cell infiltration, and glomerular crescent formation in the MRL/lpr model group. Masson staining showed more collagen deposition in the glomeruli of the MRL/lpr model group (Fig. 5*F*). Semiquantitative statistics of pathological scores showed that mesangial cell proliferation, glomerular PAS^+^ deposition, tubular lesions, crescentic lesion scores, and glomerular collagen^+^ deposition were lower in mice in the normal control group than in the MRL/lpr group (Fig. 5, *G* and *H*). qPCR results showed that the expression levels of core genes Oas2, Ifi44l, and Rnase1 in the kidneys of MRL/lpr mice were significantly higher than those in the normal control group while the expression levels of Cdt1, Eif2ak2, and Ifit3 in the kidneys of the two groups were not significantly different (Fig. 5*I*). The aforementioned experiments demonstrated that the core genes related to LN, as identified through our screening process, also exhibited aberrant expression patterns in the kidney of the mouse model.

### Human Neutrophil Activation Forms NETs and Was Positively Correlated With Core Gene Expression

**Figure 6.**
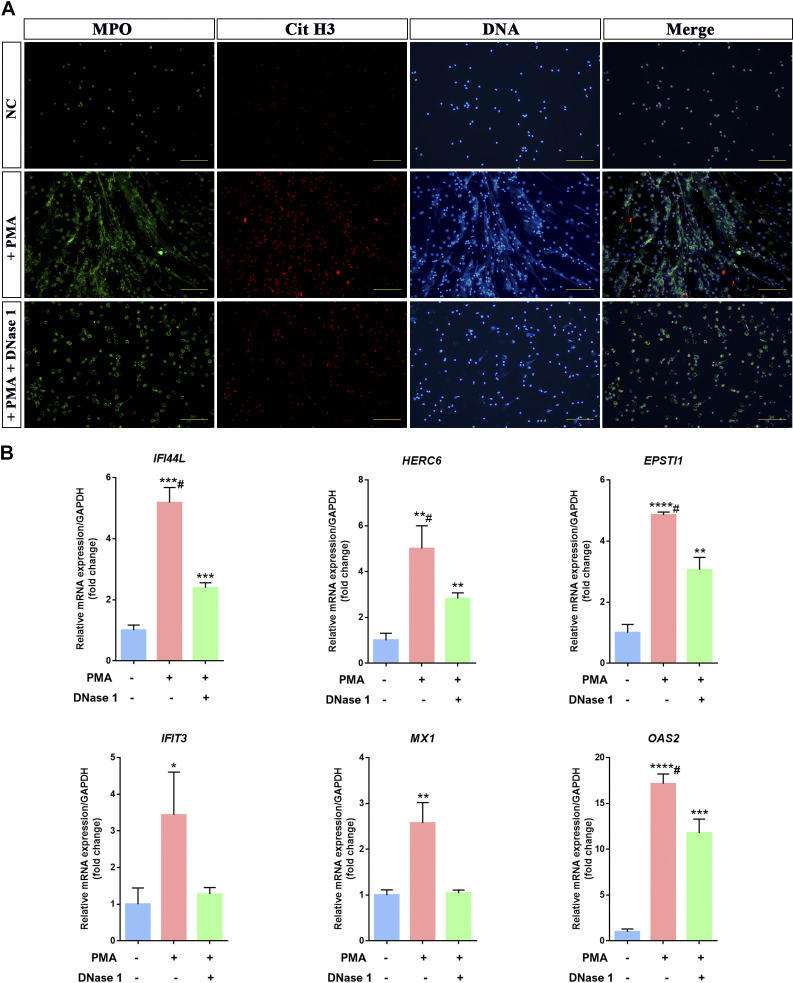
Neutrophil extracellular traps (NETs) promote core gene expression in the in vitro cell model. *A*: immunofluorescence analysis of NETs in PMA-treated neutrophils, PMA + DNase I-treated neutrophils, or NC. Bar = 100 μm. *B*: after treatment with 25 ng/mL PMA for 4 h, 25 ng/mL PMA and 1 mg/mL DNase I for 4 h, or no treatment, mRNA expression of human IFI44L, HERC6, EPSTI1, IFIT3, MX1, and OAS2 was assessed by qRT-PCR in human neutrophils (*n* = 3). Data are means ± SD for three neutrophil samples. *P < 0.05, **P < 0.01, ***P < 0.001, and ****P < 0.0001 vs. no treatment group (*t* test). #*P* < 0.05 vs. PMA-treated group (Bonferroni correction; two comparisons were made). NC, normal control.

Based on the GO analysis, neutrophil activation participated in the pathological process of LN. To further confirm whether the selected LN-related core genes were related to neutrophil activation, we isolated healthy human neutrophils and treated them with PMA to promote their activation and form NETs. PMA is an activator of protein kinase C (PKC) that can induce neutrophil activation and form neutrophil extranuclear NETs in vitro (40, 41). Immunofluorescence staining results demonstrated strongly positive expression of MPO and CitH3, which are markers for NET formation, in cells treated with PMA for 24 h compared with the normal control group. Conversely, when neutrophils were treated with PMA along with the NET inhibitor DNase I, there was a noticeable reduction in MPO and CitH3 expression (Fig. 6*A*).

We used qPCR to detect the expression changes of previously screened core genes. The results demonstrated a significant upregulation in the expression levels of core genes Ifi44l, HERC6, EPST11, Ifit3, MX1, and Oas2 in cells treated with PMA for 24 h compared with normal control neutrophils. However, upon treatment with both PMA and DNase I (Fig. 6*B*), there was a significant downregulation observed in the expression levels of these core genes. These results suggested that NETs formed after neutrophil activation can promote the upregulation of LN-related core genes, and DNase I can effectively reduce the NETs generated by neutrophil activation while inhibiting the upregulation of LN-related core genes.

### DNase I Attenuated Lupus-Like Signs in MRL/lpr Mice and Protected the Kidney

**Figure 7.**
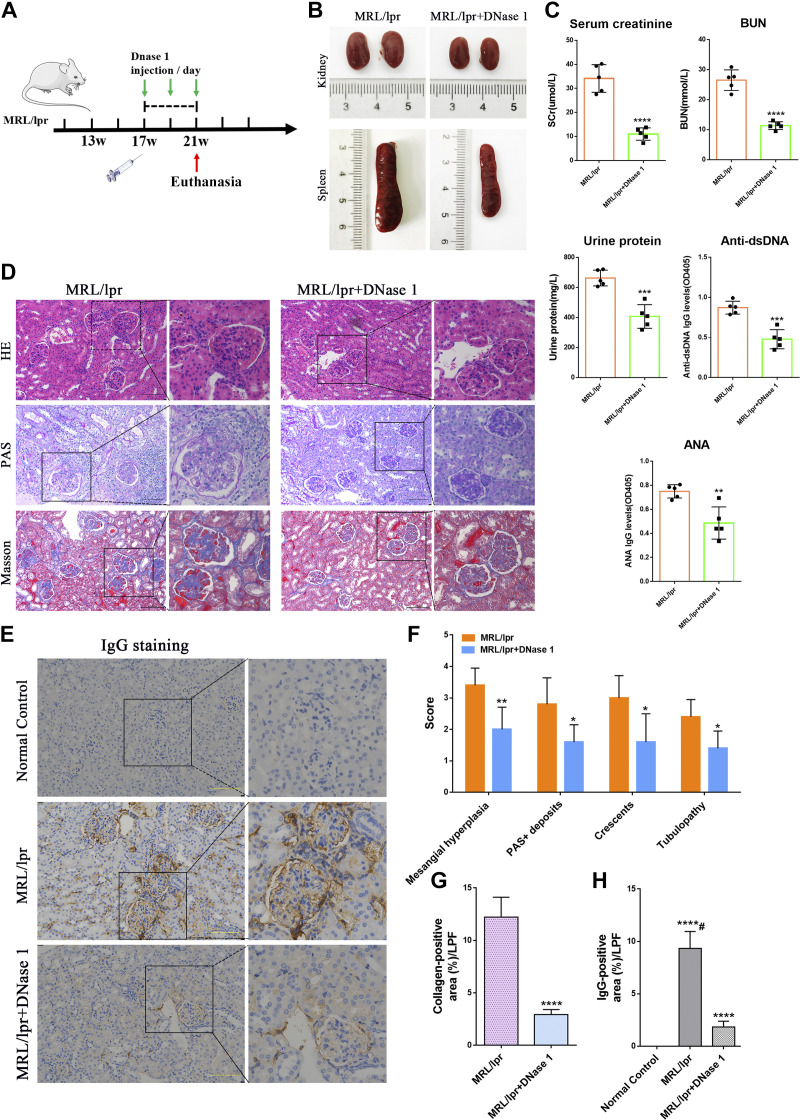
DNase I improves lupus-like signs and reduce lupus kidney pathological damage in MRL/lpr mice. *A*: strategy of DNase I administration in MRL/lpr mice. *B*: images of kidneys and spleens in the MRL/lpr group and DNase I treatment group. *C*: serum creatinine (SCr), blood urea nitrogen (BUN), urine protein, anti-double-stranded DNA antibody (Anti-dsDNA), and anti-nuclear antibody (ANA) levels in 21 wk of MRL/lpr mice and DNase I-treated mice (*n* = 5). *D*: representative images of glomerular HE, PAS, and Masson staining in the 21 wk of MRL/lpr group and DNase I treatment group. Bar = 100 μm. *E*: glomerular IgG deposition in representative renal sections from mice treated with DNase I, MRL/lpr mice, or normal control at the 21st week. Bar = 100 μm. *F*: semiquantitative pathological score in the 21 wk of MRL/lpr group and DNase I treatment group. *G*: quantification of the blue collagen-positive area (%) in both groups of mice. LPF, low-power field. *H*: bar graph presentation of the percent of IgG-positive areas in DNase I-treated mice, MRL/lpr mice, or normal control at the 21st week. Data are means ± SD for groups of five mice. *P < 0.05, **P < 0.01, ***P < 0.001, and ****P < 0.0001 vs. 21 wk of MRL/lpr mice or normal control (*t* test). #*P* < 0.05 vs. 21 wk of MRL/lpr mice (Bonferroni correction; two comparisons were made). HE, hematoxylin and eosin; PAS, periodic acid-Schiff.

MRL/lpr mice were intraperitoneally injected with DNase I from the 17th week and continued to be treated for 4 wk (Fig. 7*A*). At the 21st week, serum creatinine, serum urea, urinary protein, anti-dsDNA, and ANA concentrations were significantly decreased (Fig. 7*C*), indicating that DNase I alleviated kidney injury in MRL/lpr lupus mice. Compared with mice in the untreated MRL/lpr model group, kidney swelling and spleen enlargement were reduced in the DNase I-treated group (Fig. 7*B*).

HE and PAS staining showed glomerular immune complex deposition, renal interstitial inflammatory cell infiltration, and glomerular crescent formation in MRL/lpr mice. Mice treated with DNase I had milder glomerular damage, with only moderate mesangial and endothelial cell proliferation and less inflammatory cell infiltration (Fig. 7*D*). Masson staining showed glomerular collagen deposition in the MRL/lpr model group, while it was reduced in DNase I treatment group. Semiquantitative statistics of pathological scores showed that glomerular mesangial cell proliferation, glomerular PAS^+^ deposition, tubular lesions, crescent lesion scores (Fig. 7*F*), and glomerular collagen^+^ deposition (Fig. 7*G*) were lower in the DNase I treatment group than in the MRL/lpr group. Immunohistochemical staining results showed that compared with the normal control group (21 wk), the kidneys of the MRL/lpr model group and DNase I treatment group had a certain degree of positive expression of IgG antibody, but the positive expression of IgG antibody in the kidneys of the DNase I treatment group was significantly lower than that of the MRL/lpr model group (Fig. 7*E*). Immunohistochemical quantitative analysis showed that the proportion of IgG antibody-positive expression areas in the kidneys of mice in the DNase I-treated group was significantly lower than in the MRL/lpr model group (Fig. 7*H*). These results suggest that DNase I can alleviate renal injury and play a renoprotective role in LN mouse models.

### Degradation of NETs Downregulated the Expression of LN-Related Core Genes in MRL/lpr Mouse Kidneys

**Figure 8.**
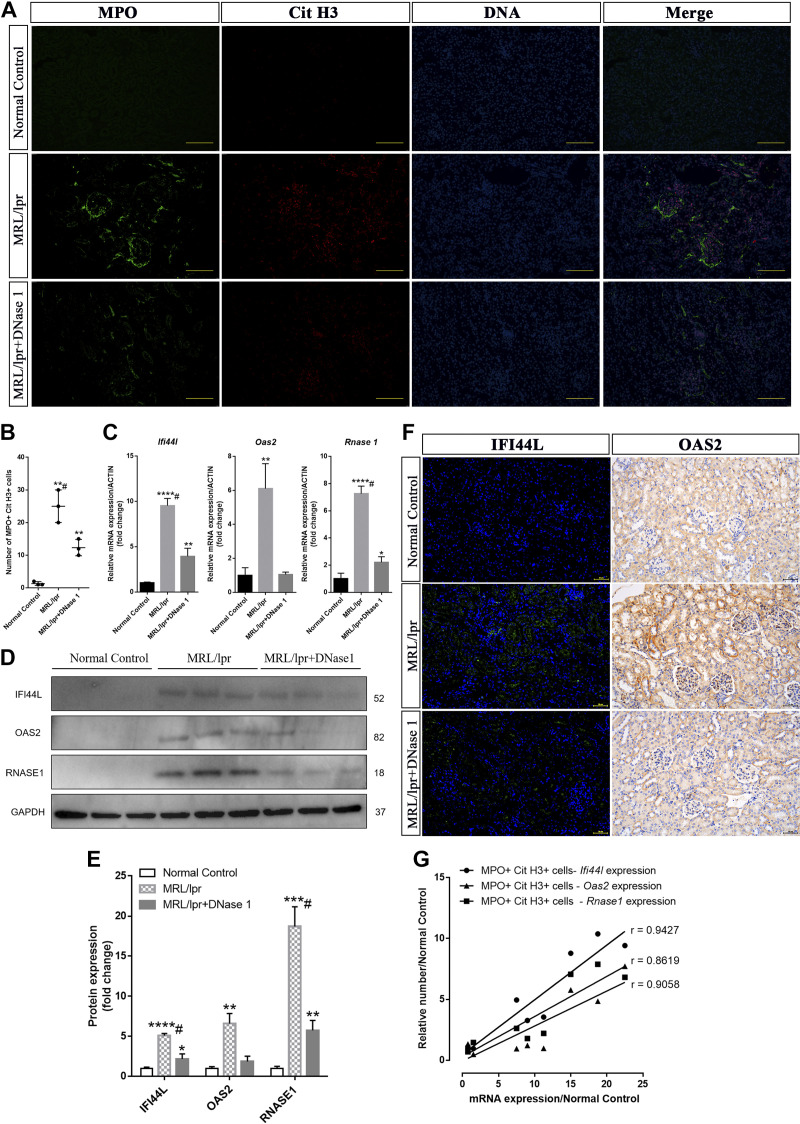
Degradation of neutrophil extracellular traps (NETs) by DNase I suppresses core genes expression. *A*: immunofluorescence analysis of NETs in representative renal sections from mice treated with DNase I, 21 wk of MRL/lpr mice, or normal control. MPO: green; Cit H3: red; DNA: blue. Bar = 100 μm. *B*: scatter diagram presentation of the numbers of MPO and Cit H3 double-positive cells in the MRL/lpr group, DNase I treatment group, and normal control (*n* = 3). *C*: mRNA expression of mouse Ifi44l, Oas2, and Rnase 1 assessed by qRT-PCR in DNase I-treated mice, MRL/lpr mice, or normal control. *D* and *E*: protein expression of IFI44L, OAS2, and RNASE1 in the MRL/lpr group, DNase I treatment group, and normal control at the 21st week as assessed by Western blotting. *F*: representative IFI44L-stained kidney sections by immunofluorescence and immunohistochemistry analysis of OAS2 from the MRL/lpr group, DNase I treatment group, and normal control mice at the 21st week. IFI44L: green; DAPI: blue. Bar = 50 μm. *G*: correlation statistical analysis between numbers of MPO and Cit H3 double-positive cells and related molecular markers (Ifi44l, *P* = 0.0001; Oas2, *P* = 0.0028; Rnase 1, *P* = 0.0008) expression in different groups of kidneys. Data are means ± SD for groups of five mice. *P < 0.05, **P < 0.01, ***P < 0.001, and ****P < 0.0001 vs. normal control (*t* test). #*P* < 0.05 vs. 21 wk of MRL/lpr mice (Bonferroni correction; two comparisons were made).

Immunofluorescence staining results of kidney sections revealed robust positive expression of MPO and CitH3, markers associated with NETs, in the kidneys of MRL/lpr mice compared with normal control mice, while the positive expression of MPO and CitH3 in the kidneys of MRL/lpr mice was significantly reduced after DNase I treatment (Fig. 8, *A* and *B*). qPCR results demonstrated that after DNase I treatment, the expressions of core genes Ifi44l, Oas2, and Rnase1 in the kidneys of MRL/lpr mice were significantly reduced compared with those of untreated MRL/lpr mice (Fig. 8*C*). Western blot analysis further confirmed that the protein expression levels of IFI44L, OAS2, and RNASE1 were also significantly decreased in the DNase I treatment group compared with the untreated MRL/lpr group (Fig. 8, *D* and *E*). Meanwhile, immunofluorescence and immunohistochemistry analysis of kidney sections revealed that IFI44L and OAS2 protein expression was present in the kidneys in MRL/lpr and MRL/lpr + DNase I-treated groups, but the expression of IFI44L and OAS2 was undetectable in the normal control group (Fig. 8*F*). Furthermore, correlation analysis indicated that the mRNA expression of these core genes was positively correlated with the number of MPO CitH3 double-positive cells (Fig. 8*G*). Ultimately, our findings demonstrated in vivo that DNase I can downregulate the expression of LN-related core genes in the kidney by reducing NETs in the kidney.

## DISCUSSION

Although the application of immunosuppressive agents and biological agents has improved the prognosis and survival rate of patients with LN, ∼10–20% of patients with LN still progress to end-stage renal disease (42). Therefore, there is an urgent need to explore novel therapeutic approaches aimed at preventing LN progression and prolonging patient survival. In this study, we used bioinformatics joint analysis to identify a total of 976 DEGs in PBMCs and 2,427 DEGs in kidney samples between LN and normal samples, respectively. Ultimately, we screened out 16 core genes shared by PBMCs and kidney tissue that were closely associated with LN. Molecular biological methods were used to verify the abnormal expression of these core genes in both PBMCs of patients with LN and the mouse model, and mechanism experiments showed that these core genes were related to NETs formed by neutrophil activation. Targeted degradation of NETs could downregulate the expression levels of these core genes and alleviate kidney injury in LN mouse models.

To gain a deeper understanding of the progression of LN, we used WGCNA to identify the key modules associated with LN. Through GO and KEGG pathway enrichment analysis of DEGs within these positively correlated modules, we found that innate/adaptive immune responses and cell proliferation, particularly in neutrophil activation pathways, are heightened active in both PBMCs and the kidney. However, analysis of the number of immune cells in the patient’s PBMCs and kidney tissue showed that the number of neutrophils did not increase significantly in either, while there was a notable increase in the number of monocytes. These results were consistent with previous reports indicating upregulation of neutrophil-related gene expression in patients with SLE without a significant increase in neutrophil count (43). One potential explanation for the lack of significant changes in neutrophil count could be attributed to treatment interventions, such as the administration of glucocorticoids (GCs), which are commonly used in cases of LN. It is worth noting that the differences in peripheral blood neutrophils observed between patients with LN and patients with non-LN SLE disappeared following GC therapy, indicating that GC usage may serve as a prominent confounder contributing to the mismatch between neutrophil activation and cell count (43). In addition, it has been previously reported that GCs can enhance the proportion of immature cells in neutrophils. Among these, low-density granulocytes (LDGs) may represent an activated population of immature neutrophils due to their less segmented and more lobular nature (44, 45). This may be the main reason that GC therapy promotes the expression of neutrophil-associated genes in LN, while neutrophil counts do not change significantly. Moreover, it has been found that neutrophil-specific genes were highly expressed in PBMCs of patients with lupus, and further investigations have confirmed the presence of a subset of LDGs within the monocyte fraction, which is activated and triggers autoimmune responses and organ damage in SLE (46, 47). Further research of mechanism indicated that the pathogenic role of this type of neutrophils is exerted through injury to endothelial cells and increased levels of proinflammatory cytokines and type I IFN (45). Consequently, in both PBMCs and kidneys of patients with LN, the DEGs significant enriched in pathways associated with neutrophil activation due to the presence of these LDGs. As a result, there was an increase in the number of monocytes while the number of conventional neutrophils may not change significantly.

Although the potential role of neutrophils in LN was suggested decades ago (48), it remains unclear whether neutrophils play a pathogenic role in kidney injury associated with LN. The latest study has confirmed the presence of NETs in the kidney through kidney biopsy from patients with LN, especially in the inflammatory glomerular area, and the number of NET infiltration was found to be positively correlated with pathological symptoms of LN (38). NETs are a unique form of neutrophil death characterized by a chromatin fiber extracellular network structure composed of immune-stimulating molecules and various enzymes and proteins from the cytoplasm and neutrophil particles (21, 49). They can stimulate the synthesis of type I IFN and proinflammatory cytokines by immune cells to induce kidney injury (26, 45, 50). In this study, we confirmed that NETs were indeed present in the kidney of the LN mouse model, and combined with the in vitro neutrophil activation induction model, it was found to be positively correlated with abnormal expression of the screened LN core gene.

Studies have demonstrated the widespread presence of NETs in the kidneys of patients with autoimmune diseases, which can facilitate immune complex formation within the renal tissue (25). In-depth investigations have indicated that impaired degradation of NETs plays a crucial role in the progression of lupus nephritis, as DNase I inhibitors and anti-NET antibodies hinder DNase I-mediated breakdown of NETs (38). Furthermore, mutations in the DNase I gene, an essential molecule responsible for NET degradation, represent another pivotal factor contributing to NET accumulation, and it can lead to severe lupus-like syndrome and the occurrence of LN (51). Hence, these findings suggest that targeted clearance of NETs could be beneficial for achieving disease remission in LN. In our study, application of recombinant DNase I significantly reduced both activated NETs in vitro and NETs in mouse model kidneys and mitigated kidney injury in mice. Remarkably, the abnormal expression of the core genes obtained by screening was somewhat corrected following the reduction of NET levels.

As two signaling pathways mediated by innate immune cells, neutrophil activation and the type I IFN pathway play a crucial role in LN, with a close interrelationship between them (38, 52). It has been previously reported that NETs generated through neutrophil activation in LN significantly enhance the secretion of IFN-α by dendritic cells, while inhibition of NETs reduces IFN-α production (53). Furthermore, in SLE studies, it has been confirmed that NETs induced by immune complexes can stimulate the secretion of IFN-α by plasmacytoid dendritic cells strongly through binding to IL-33 (54). The majority of the 16 core genes identified and obtained in this study were associated with the type I IFN pathway, including CMPK2 (55), OAS2 (56), PLSCR1 (57), IFI44L (58), HERC6 (59), DDX60 (60), EPSTI1 (61), EIF2AK2 (62), IFIT3 (63), and MX1 (64). Therefore, when NETs were degraded by DNase I, the type I IFN pathway was inhibited, and the expression of these related genes must be reduced. In addition, the core genes (including CCNA2, TOP2A, CDT1, HSPA8, and TSG101) were also enriched in cell cycle regulatory signaling pathways, which was consistent with the recent findings of enhanced cell cycle signaling pathways in patients with LN and SLE revealed by high-pathway sequencing (65, 66). Therefore, with the improvement of pathological symptoms in animal models after treatment with DNase I, core genes related to the cell cycle are also downregulated.

LN is a multifactorial disease resulting from the interplay of multiple susceptibility genes. In recent years, an increasing number of genes have been identified as being associated with LN (5, 16, 67). Neutrophil activation, which involves several potential candidate genes, plays a pivotal role in the pathogenesis of LN (68). In this study, we screened and identified 16 core genes that may play a role in neutrophil activation, and the expression of some genes was increased in PBMCs of patients with active renal involvement and in mouse model kidneys. However, the expressions of some genes (Cdt1, Eif2ak2, and Ifit3) were upregulated but showed no significant differences. First, this may be attributed to species differences. Second, research reports have indicated that the expression patterns of some cytokines (IL-2, IL-5, IL-6, etc.) are significantly different between the MRL/lpr mouse model and clinical patients with LN (69, 70), suggesting that the MRL/lpr mouse model, as one of the models of spontaneous lupus, may not fully simulate the whole pathophysiological process of human LN disease. Furthermore, other studies using bioinformatic analysis showed similar situations to our results, such as transcriptional network analysis of cross-species inflammatory response in mice and human LN. It was found that the gene expression of endoplasmic reticulum stress, hypoxia response, and downregulated acute inflammation pathway molecules were inconsistent in mouse models and human samples (71). Similarly, in a genetic screening study using bioinformatics from a public database of patients with LN and mouse lupus models, only a small number of overlapping differential genes were screened (72).

IFI44L has been recognized as an indicator of IFN signaling, and its hypomethylation has been linked to increased susceptibility to SLE (73). Recent studies have reported a significant upregulation of IFI44L gene expression in peripheral blood and synovial tissues of patients with SLE (58, 74). Our findings demonstrate aberrant expression of IFI44L closely associated with neutrophil activation in both patients with LN and mouse models. OAS2, as a member of the OAS family, also plays an important role in LN development (11). Furthermore, previous research has indicated a positive correlation between OAS gene expression and the infiltration of immune cells (especially neutrophils, dendritic cells, etc.) (75). We also found that OAS2 expression was upregulated with an increase of NETs formed by neutrophil infiltration in the kidney of the LN mouse model and downregulated with the degradation of NETs. RNASE1 belongs to the ribonuclease A superfamily, and recent evidence suggests its potential to modulate immune cell infiltration, thereby affecting the immune function in patients with SLE (76). The expression of RNase1 was also observed to increase in the synovium of rheumatoid arthritis (77). In a study related to angiogenesis, it was found that RNASE1 inhibits angiogenesis by reducing the formation of NETs (78). This result is contrary to the positive correlation between increased NETs and RNASE1 expression in the kidney in our study. We suggested that the increase of RNASE1 in the kidney may be the result of increased NET formation, reflecting an anti-inflammatory attempt to reduce the proinflammatory effect of NETs, and that the downregulation of RNASE1 expression after degradation of NETs by DNase I is a similar compensatory mechanism. The aberrant expressions of these core genes were regulated by distinct biological pathways, with neutrophil activation potentially playing an important role. However, the key functional roles that these core genes may play in the abnormal expression of LN need to be further investigated.

In conclusion, through bioinformatics joint analysis of DEGs in PBMCs and kidney samples from patients with LN, we revealed that neutrophil activation, the type I IFN pathway, and cell cycle regulation were abnormal, and 16 core genes associated with LN were screened. Subsequently, we confirmed that the abnormal expression of these core genes was closely linked to the production of NETs, in both an in vitro cell model and an in vivo animal model. Furthermore, we found that degradation of NETs by DNase I could downregulate the expression of these core genes and improve the pathological symptoms observed in LN. Therefore, targeting neutrophil activation, particularly NET production, and inhibiting these aberrant genes represent potential therapeutic approaches for treating LN.

## DATA AVAILABILITY

The original contributions presented in the study are included in the article/Supplementary Material; further inquiries can be directed to the corresponding author.

## SUPPLEMENTAL DATA

10.6084/m9.figshare.26494903.v1 Supplemental Tables S1–S4 and Supplemental Figs. S1 and S2: https://doi.org/10.6084/m9.figshare.26494903.v1.

## GRANTS

This work was supported by the National Natural Science Foundation of China (82260138, 82260327, and 82360140); Natural Science Foundation of Inner Mongolia (2022QN08022); The Scientific Research Project of Colleges and Universities, Inner Mongolia Department of Education (NJZZ22678); Program for Young Talents of Science and Technology in Universities of Inner Mongolia Autonomous Region (NJYT23049); The Inner Mongolia Health Commission’s Medical and Health Science and Technology Program (Class A) (202201267); and the Natural Science Foundation of Inner Mongolia Medical University (YKD2022MS010).

## DISCLOSURES

No conflicts of interest, financial or otherwise, are declared by the authors.

## AUTHOR CONTRIBUTIONS

Y.J., H.L., and M.Z. conceived and designed research; Y.J., Y.W., X.M., and M.Z. performed experiments; Y.J., Y.W., X.M., and M.Z. analyzed data; Y.J. and M.Z. interpreted results of experiments; Y.J., Y.W., and X.M. prepared figures; Y.J. and M.Z. drafted manuscript; Y.J., H.L., and M.Z. edited and revised manuscript; H.L. and M.Z. approved final version of manuscript.

## References

[1] Bernatsky S, Boivin JF, Joseph L, Manzi S, Ginzler E, Gladman DD, , et al Mortality in systemic lupus erythematosus. Arthritis Rheum 54: 2550–2557, 2006. doi:10.1002/art.21955. 16868977

[2] Maria NI, Davidson A. Protecting the kidney in systemic lupus erythematosus: from diagnosis to therapy. Nat Rev Rheumatol 16: 255–267, 2020. doi:10.1038/s41584-020-0401-9. 32203285

[3] Clynes R, Dumitru C, Ravetch JV. Uncoupling of immune complex formation and kidney damage in autoimmune glomerulonephritis. Science 279: 1052–1054, 1998. doi:10.1126/science.279.5353.1052. 9461440

[4] Janeway CA Jr, Medzhitov R. Innate immune recognition. Annu Rev Immunol 20: 197–216, 2002. doi:10.1146/annurev.immunol.20.083001.084359. 11861602

[5] Yao M, Gao C, Zhang C, Di X, Liang W, Sun W, Wang Q, Zheng Z. Identification of molecular markers associated with the pathophysiology and treatment of lupus nephritis based on integrated transcriptome analysis. Front Genet 11: 583629, 2020. doi:10.3389/fgene.2020.583629. 33384713 PMC7770169

[6] Chen Z, Lan R, Ye K, Chen H, Chen C, Xu Y. Prioritization of diagnostic and prognostic biomarkers for lupus nephritis based on integrated bioinformatics analyses. Front Bioeng Biotechnol 9: 717234, 2021. doi:10.3389/fbioe.2021.717234. 34692653 PMC8531593

[7] Arazi A, Rao DA, Berthier CC, Davidson A, Liu Y, Hoover PJ, , et al The immune cell landscape in kidneys of patients with lupus nephritis. Nat Immunol 20: 902–914, 2019. doi:10.1038/s41590-019-0398-x. 31209404 PMC6726437

[8] Dong Z, Dai H, Liu W, Jiang H, Feng Z, Liu F, Zhao Q, Rui H, Liu WJ, Liu B. Exploring the differences in molecular mechanisms and key biomarkers between membranous nephropathy and lupus nephritis using integrated bioinformatics analysis. Front Genet 12: 770902, 2021. doi:10.3389/fgene.2021.770902. 35047003 PMC8762271

[9] Woroniecka KI, Park AS, Mohtat D, Thomas DB, Pullman JM, Susztak K. Transcriptome analysis of human diabetic kidney disease. Diabetes 60: 2354–2369, 2011. doi:10.2337/db10-1181. 21752957 PMC3161334

[10] Ouzounis CA, Valencia A. Early bioinformatics: the birth of a discipline–a personal view. Bioinformatics 19: 2176–2190, 2003. doi:10.1093/bioinformatics/btg309. 14630646

[11] Cao Y, Mi X, Wang Z, Zhang D, Tang W. Bioinformatic analysis reveals that the OAS family may play an important role in lupus nephritis. J Natl Med Assoc 112: 567–577, 2020. doi:10.1016/j.jnma.2020.05.006. 32622555

[12] Cao Y, Tang W, Tang W. Immune cell infiltration characteristics and related core genes in lupus nephritis: results from bioinformatic analysis. BMC Immunol 20: 37, 2019. doi:10.1186/s12865-019-0316-x. 31638917 PMC6805654

[13] Nikpour M, Dempsey AA, Urowitz MB, Gladman DD, Barnes DA. Association of a gene expression profile from whole blood with disease activity in systemic lupus erythematosus. Ann Rheum Dis 67: 1069–1075, 2008. doi:10.1136/ard.2007.074765. 18063674

[14] Baechler EC, Batliwalla FM, Karypis G, Gaffney PM, Ortmann WA, Espe KJ, Shark KB, Grande WJ, Hughes KM, Kapur V, Gregersen PK, Behrens TW. Interferon-inducible gene expression signature in peripheral blood cells of patients with severe lupus. Proc Natl Acad Sci USA 100: 2610–2615, 2003. doi:10.1073/pnas.0337679100. 12604793 PMC151388

[15] Zhu H, Mi W, Luo H, Chen T, Liu S, Raman I, Zuo X, Li QZ. Whole-genome transcription and DNA methylation analysis of peripheral blood mononuclear cells identified aberrant gene regulation pathways in systemic lupus erythematosus. Arthritis Res Ther 18: 162, 2016. doi:10.1186/s13075-016-1050-x. 27412348 PMC4942934

[16] Zhang W, Liang G, Zhou H, Zeng X, Zhang Z, Xu X, Lai K. Identification of potential biomarkers for systemic lupus erythematosus by integrated analysis of gene expression and methylation data. Clin Rheumatol 42: 1423–1433, 2023. doi:10.1007/s10067-022-06495-3. 36595110

[17] Iwata Y, Bostrom EA, Menke J, Rabacal WA, Morel L, Wada T, Kelley VR. Aberrant macrophages mediate defective kidney repair that triggers nephritis in lupus-susceptible mice. J Immunol 188: 4568–4580, 2012. doi:10.4049/jimmunol.1102154. 22467656 PMC3340928

[18] Chen PM, Wilson PC, Shyer JA, Veselits M, Steach HR, Cui C, Moeckel G, Clark MR, Craft J. Kidney tissue hypoxia dictates T cell-mediated injury in murine lupus nephritis. Sci Transl Med 12: eaay1620, 2020. doi:10.1126/scitranslmed.aay1620. 32269165 PMC8055156

[19] Jing C, Castro-Dopico T, Richoz N, Tuong ZK, Ferdinand JR, Lok LSC, Loudon KW, Banham GD, Mathews RJ, Cader Z, Fitzpatrick S, Bashant KR, Kaplan MJ, Kaser A, Johnson RS, Murphy MP, Siegel RM, Clatworthy MR. Macrophage metabolic reprogramming presents a therapeutic target in lupus nephritis. Proc Natl Acad Sci USA 117: 15160–15171, 2020. doi:10.1073/pnas.2000943117. 32541026 PMC7334513

[20] Masucci MT, Minopoli M, Del Vecchio S, Carriero MV. The emerging role of neutrophil extracellular traps (NETs) in tumor progression and metastasis. Front Immunol 11: 1749, 2020. doi:10.3389/fimmu.2020.01749. 33042107 PMC7524869

[21] Brinkmann V, Reichard U, Goosmann C, Fauler B, Uhlemann Y, Weiss DS, Weinrauch Y, Zychlinsky A. Neutrophil extracellular traps kill bacteria. Science 303: 1532–1535, 2004. doi:10.1126/science.1092385. 15001782

[22] Garishah FM, Rother N, Riswari SF, Alisjahbana B, Overheul GJ, van Rij RP, van der Ven A, van der Vlag J, de Mast Q. Neutrophil extracellular traps in dengue are mainly generated NOX-independently. Front Immunol 12: 629167, 2021. doi:10.3389/fimmu.2021.629167. 34122402 PMC8187769

[23] Nakazawa D, Marschner JA, Platen L, Anders HJ. Extracellular traps in kidney disease. Kidney Int 94: 1087–1098, 2018. doi:10.1016/j.kint.2018.08.035. 30466565

[24] Gupta S, Kaplan MJ. The role of neutrophils and NETosis in autoimmune and renal diseases. Nat Rev Nephrol 12: 402–413, 2016. doi:10.1038/nrneph.2016.71. 27241241 PMC5510606

[25] Kessenbrock K, Krumbholz M, Schonermarck U, Back W, Gross WL, Werb Z, Grone HJ, Brinkmann V, Jenne DE. Netting neutrophils in autoimmune small-vessel vasculitis. Nat Med 15: 623–625, 2009. doi:10.1038/nm.1959. 19448636 PMC2760083

[26] Garcia-Romo GS, Caielli S, Vega B, Connolly J, Allantaz F, Xu Z, Punaro M, Baisch J, Guiducci C, Coffman RL, Barrat FJ, Banchereau J, Pascual V. Netting neutrophils are major inducers of type I IFN production in pediatric systemic lupus erythematosus. Sci Transl Med 3: 73ra20, 2011. doi:10.1126/scitranslmed.3001201. 21389264 PMC3143837

[27] Lande R, Ganguly D, Facchinetti V, Frasca L, Conrad C, Gregorio J, Meller S, Chamilos G, Sebasigari R, Riccieri V, Bassett R, Amuro H, Fukuhara S, Ito T, Liu YJ, Gilliet M. Neutrophils activate plasmacytoid dendritic cells by releasing self-DNA-peptide complexes in systemic lupus erythematosus. Sci Transl Med 3: 73ra19, 2011. doi:10.1126/scitranslmed.3001180. 21389263 PMC3399524

[28] Deng Y, Zheng Y, Li D, Hong Q, Zhang M, Li Q, Fu B, Wu L, Wang X, Shen W, Zhang Y, Chang J, Song K, Liu X, Shang S, Cai G, Chen X. Expression characteristics of interferon-stimulated genes and possible regulatory mechanisms in lupus patients using transcriptomics analyses. EBioMedicine 70: 103477, 2021. doi:10.1016/j.ebiom.2021.103477. 34284174 PMC8318865

[29] Langfelder P, Horvath S. WGCNA: an R package for weighted correlation network analysis. BMC Bioinformatics 9: 559, 2008. doi:10.1186/1471-2105-9-559. 19114008 PMC2631488

[30] Ali HR, Chlon L, Pharoah PD, Markowetz F, Caldas C. Patterns of immune infiltration in breast cancer and their clinical implications: a gene-expression-based retrospective study. PLoS Med 13: e1002194, 2016. doi:10.1371/journal.pmed.1002194. 27959923 PMC5154505

[31] Panousis NI, Bertsias GK, Ongen H, Gergianaki I, Tektonidou MG, Trachana M, Romano-Palumbo L, Bielser D, Howald C, Pamfil C, Fanouriakis A, Kosmara D, Repa A, Sidiropoulos P, Dermitzakis ET, Boumpas DT. Combined genetic and transcriptome analysis of patients with SLE: distinct, targetable signatures for susceptibility and severity. Ann Rheum Dis 78: 1079–1089, 2019. doi:10.1136/annrheumdis-2018-214379. 31167757 PMC6691930

[32] Newman AM, Liu CL, Green MR, Gentles AJ, Feng W, Xu Y, Hoang CD, Diehn M, Alizadeh AA. Robust enumeration of cell subsets from tissue expression profiles. Nat Methods 12: 453–457, 2015. doi:10.1038/nmeth.3337. 25822800 PMC4739640

[33] Song E, Song W, Ren M, Xing L, Ni W, Li Y, Gong M, Zhao M, Ma X, Zhang X, An R. Identification of potential crucial genes associated with carcinogenesis of clear cell renal cell carcinoma. J Cell Biochem 119: 5163–5174, 2018. doi:10.1002/jcb.26543. 29227586

[34] Bader GD, Hogue CW. An automated method for finding molecular complexes in large protein interaction networks. BMC Bioinformatics 4: 2, 2003. doi:10.1186/1471-2105-4-2. 12525261 PMC149346

[35] Macanovic M, Sinicropi D, Shak S, Baughman S, Thiru S, Lachmann PJ. The treatment of systemic lupus erythematosus (SLE) in NZB/W F1 hybrid mice; studies with recombinant murine DNase and with dexamethasone. Clin Exp Immunol 106: 243–252, 1996. doi:10.1046/j.1365-2249.1996.d01-839.x. 8918569 PMC2200591

[36] Wang H, Lu M, Zhai S, Wu K, Peng L, Yang J, Xia Y. ALW peptide ameliorates lupus nephritis in MRL/lpr mice. Arthritis Res Ther 21: 261, 2019. doi:10.1186/s13075-019-2038-0. 31791413 PMC6889545

[37] Saisorn W, Saithong S, Phuengmaung P, Udompornpitak K, Bhunyakarnjanarat T, Visitchanakun P, Chareonsappakit A, Pisitkun P, Chiewchengchol D, Leelahavanichkul A. Acute kidney injury induced lupus exacerbation through the enhanced neutrophil extracellular traps (and apoptosis) in Fcgr2b deficient lupus mice with renal ischemia reperfusion injury. Front Immunol 12: 669162, 2021. doi:10.3389/fimmu.2021.669162. 34248948 PMC8269073

[38] Hakkim A, Furnrohr BG, Amann K, Laube B, Abed UA, Brinkmann V, Herrmann M, Voll RE, Zychlinsky A. Impairment of neutrophil extracellular trap degradation is associated with lupus nephritis. Proc Natl Acad Sci USA 107: 9813–9818, 2010. doi:10.1073/pnas.0909927107. 20439745 PMC2906830

[39] Jin Y, Zhang M, Li M, Zhang H, Zhao L, Qian C, Li S, Zhang H, Gao M, Pan B, Li R, Wan X, Cao C. SIX1 activation is involved in cell proliferation, migration, and anti-inflammation of acute ischemia/reperfusion injury in mice. Front Mol Biosci 8: 725319, 2021. doi:10.3389/fmolb.2021.725319. 34513929 PMC8427868

[40] Han F, Ding ZF, Shi XL, Zhu QT, Shen QH, Xu XM, Zhang JX, Gong WJ, Xiao WM, Wang D, Chen WW, Hu LH, Lu GT. Irisin inhibits neutrophil extracellular traps formation and protects against acute pancreatitis in mice. Redox Biol 64: 102787, 2023. doi:10.1016/j.redox.2023.102787. 37392517 PMC10336674

[41] Bruschi M, Petretto A, Santucci L, Vaglio A, Pratesi F, Migliorini P, Bertelli R, Lavarello C, Bartolucci M, Candiano G, Prunotto M, Ghiggeri GM. Neutrophil extracellular traps protein composition is specific for patients with Lupus nephritis and includes methyl-oxidized alphaenolase (methionine sulfoxide 93). Sci Rep 9: 7934, 2019. doi:10.1038/s41598-019-44379-w. 31138830 PMC6538718

[42] Gomez-Puerta JA, Feldman CH, Alarcon GS, Guan H, Winkelmayer WC, Costenbader KH. Racial and ethnic differences in mortality and cardiovascular events among patients with end-stage renal disease due to lupus nephritis. Arthritis Care Res (Hoboken) 67: 1453–1462, 2015. doi:10.1002/acr.22562. 25624071 PMC4515402

[43] Wither JE, Prokopec SD, Noamani B, Chang NH, Bonilla D, Touma Z, Avila-Casado C, Reich HN, Scholey J, Fortin PR, Boutros PC, Landolt-Marticorena C. Identification of a neutrophil-related gene expression signature that is enriched in adult systemic lupus erythematosus patients with active nephritis: clinical/pathologic associations and etiologic mechanisms. PLoS One 13: e0196117, 2018. doi:10.1371/journal.pone.0196117. 29742110 PMC5942792

[44] Denny MF, Yalavarthi S, Zhao W, Thacker SG, Anderson M, Sandy AR, McCune WJ, Kaplan MJ. A distinct subset of proinflammatory neutrophils isolated from patients with systemic lupus erythematosus induces vascular damage and synthesizes type I IFNs. J Immunol 184: 3284–3297, 2010 [Erratum in J Immunol 185: 3779, 2010]. doi:10.4049/jimmunol.0902199. 20164424 PMC2929645

[45] Villanueva E, Yalavarthi S, Berthier CC, Hodgin JB, Khandpur R, Lin AM, Rubin CJ, Zhao W, Olsen SH, Klinker M, Shealy D, Denny MF, Plumas J, Chaperot L, Kretzler M, Bruce AT, Kaplan MJ. Netting neutrophils induce endothelial damage, infiltrate tissues, and expose immunostimulatory molecules in systemic lupus erythematosus. J Immunol 187: 538–552, 2011. doi:10.4049/jimmunol.1100450. 21613614 PMC3119769

[46] Bennett L, Palucka AK, Arce E, Cantrell V, Borvak J, Banchereau J, Pascual V. Interferon and granulopoiesis signatures in systemic lupus erythematosus blood. J Exp Med 197: 711–723, 2003. doi:10.1084/jem.20021553. 12642603 PMC2193846

[47] Hacbarth E, Kajdacsy-Balla A. Low density neutrophils in patients with systemic lupus erythematosus, rheumatoid arthritis, and acute rheumatic fever. Arthritis Rheum 29: 1334–1342, 1986. doi:10.1002/art.1780291105. 2430586

[48] Camussi G, Cappio FC, Messina M, Coppo R, Stratta P, Vercellone A. The polymorphonuclear neutrophil (PMN) immunohistological technique: detection of immune complexes bound to the PMN membrane in acute poststreptococcal and lupus nephritis. Clin Nephrol 14: 280–287, 1980. 7008994

[49] Fuchs TA, Abed U, Goosmann C, Hurwitz R, Schulze I, Wahn V, Weinrauch Y, Brinkmann V, Zychlinsky A. Novel cell death program leads to neutrophil extracellular traps. J Cell Biol 176: 231–241, 2007. doi:10.1083/jcb.200606027. 17210947 PMC2063942

[50] Lood C, Blanco LP, Purmalek MM, Carmona-Rivera C, De Ravin SS, Smith CK, Malech HL, Ledbetter JA, Elkon KB, Kaplan MJ. Neutrophil extracellular traps enriched in oxidized mitochondrial DNA are interferogenic and contribute to lupus-like disease. Nat Med 22: 146–153, 2016. doi:10.1038/nm.4027. 26779811 PMC4742415

[51] Yasutomo K, Horiuchi T, Kagami S, Tsukamoto H, Hashimura C, Urushihara M, Kuroda Y. Mutation of DNASE1 in people with systemic lupus erythematosus. Nat Genet 28: 313–314, 2001. doi:10.1038/91070. 11479590

[52] Knight JS, Subramanian V, O'Dell AA, Yalavarthi S, Zhao W, Smith CK, Hodgin JB, Thompson PR, Kaplan MJ. Peptidylarginine deiminase inhibition disrupts NET formation and protects against kidney, skin and vascular disease in lupus-prone MRL/lpr mice. Ann Rheum Dis 74: 2199–2206, 2015. doi:10.1136/annrheumdis-2014-205365. 25104775 PMC4320672

[53] Wang H, Li T, Chen S, Gu Y, Ye S. Neutrophil extracellular trap mitochondrial DNA and its autoantibody in systemic lupus erythematosus and a proof-of-concept trial of metformin. Arthritis Rheumatol 67: 3190–3200, 2015. doi:10.1002/art.39296. 26245802

[54] Georgakis S, Gkirtzimanaki K, Papadaki G, Gakiopoulou H, Drakos E, Eloranta ML, Makridakis M, Kontostathi G, Zoidakis J, Baira E, Ronnblom L, Boumpas DT, Sidiropoulos P, Verginis P, Bertsias G. NETs decorated with bioactive IL-33 infiltrate inflamed tissues and induce IFN-alpha production in patients with SLE. JCI Insight 6: e147671, 2021. doi:10.1172/jci.insight.147671. 34554930 PMC8663547

[55] Lai JH, Hung LF, Huang CY, Wu DW, Wu CH, Ho LJ. Mitochondrial protein CMPK2 regulates IFN alpha-enhanced foam cell formation, potentially contributing to premature atherosclerosis in SLE. Arthritis Res Ther 23: 120, 2021. doi:10.1186/s13075-021-02470-6. 33874983 PMC8054390

[56] Shen M, Duan C, Xie C, Wang H, Li Z, Li B, Wang T. Identification of key interferon-stimulated genes for indicating the condition of patients with systemic lupus erythematosus. Front Immunol 13: 962393, 2022. doi:10.3389/fimmu.2022.962393. 35967341 PMC9365928

[57] Kusano S, Ikeda M. Interaction of phospholipid scramblase 1 with the Epstein-Barr virus protein BZLF1 represses BZLF1-mediated lytic gene transcription. J Biol Chem 294: 15104–15116, 2019. doi:10.1074/jbc.RA119.008193. 31434743 PMC6791327

[58] Zhao M, Zhou Y, Zhu B, Wan M, Jiang T, Tan Q, Liu Y, Jiang J, Luo S, Tan Y, Wu H, Renauer P, Del Mar Ayala Gutierrez M, Castillo Palma MJ, Ortega Castro R, Fernandez-Roldan C, Raya E, Faria R, Carvalho C, Alarcon-Riquelme ME, Xiang Z, Chen J, Li F, Ling G, Zhao H, Liao X, Lin Y, Sawalha AH, Lu Q. IFI44L promoter methylation as a blood biomarker for systemic lupus erythematosus. Ann Rheum Dis 75: 1998–2006, 2016. doi:10.1136/annrheumdis-2015-208410. 26787370 PMC4955646

[59] Uppala R, Sarkar MK, Young KZ, Ma F, Vemulapalli P, Wasikowski R, Plazyo O, Swindell WR, Maverakis E, Gharaee-Kermani M, Billi AC, Tsoi LC, Kahlenberg JM, Gudjonsson JE. HERC6 regulates STING activity in a sex-biased manner through modulation of LATS2/VGLL3 Hippo signaling. iScience 27: 108986, 2024. doi:10.1016/j.isci.2024.108986. 38327798 PMC10847730

[60] Karasawa T, Sato R, Imaizumi T, Hashimoto S, Fujita M, Aizawa T, Tsugawa K, Kawaguchi S, Seya K, Terui K, Tanaka H. Glomerular endothelial expression of type I IFN-stimulated gene, DExD/H-Box helicase 60 via toll-like receptor 3 signaling: possible involvement in the pathogenesis of lupus nephritis. Ren Fail 44: 137–145, 2022. doi:10.1080/0886022X.2022.2027249. 35392757 PMC9004514

[61] Cooles FAH, Tarn J, Lendrem DW, Naamane N, Lin CM, Millar B, Maney NJ, Anderson AE, Thalayasingam N, Diboll J, Bondet V, Duffy D, Barnes MR, Smith GR, Ng S, Watson D, Henkin R, Cope AP, Reynard LN, Pratt AG; RA-MAP Consortium; Isaacs JD. Interferon-alpha-mediated therapeutic resistance in early rheumatoid arthritis implicates epigenetic reprogramming. Ann Rheum Dis 81: 1214–1223, 2022. doi:10.1136/annrheumdis-2022-222370. 35680389 PMC9380486

[62] Fert I, Cagnard N, Glatigny S, Letourneur F, Jacques S, Smith JA, Colbert RA, Taurog JD, Chiocchia G, Araujo LM, Breban M. Reverse interferon signature is characteristic of antigen-presenting cells in human and rat spondyloarthritis. Arthritis Rheumatol 66: 841–851, 2014. doi:10.1002/art.38318. 24757137 PMC4226235

[63] Bodewes ILA, Huijser E, van Helden-Meeuwsen CG, Tas L, Huizinga R, Dalm V, van Hagen PM, Groot N, Kamphuis S, van Daele PLA, Versnel MA. TBK1: A key regulator and potential treatment target for interferon positive Sjogren's syndrome, systemic lupus erythematosus and systemic sclerosis. J Autoimmun 91: 97–102, 2018. doi:10.1016/j.jaut.2018.02.001. 29673738

[64] Feng X, Wu H, Grossman JM, Hanvivadhanakul P, FitzGerald JD, Park GS, Dong X, Chen W, Kim MH, Weng HH, Furst DE, Gorn A, McMahon M, Taylor M, Brahn E, Hahn BH, Tsao BP. Association of increased interferon-inducible gene expression with disease activity and lupus nephritis in patients with systemic lupus erythematosus. Arthritis Rheum 54: 2951–2962, 2006. doi:10.1002/art.22044. 16947629

[65] Zou X, Yang M, Ye Z, Li T, Jiang Z, Xia Y, Tan S, Long Y, Wang X. Uncovering lupus nephritis-specific genes and the potential of TNFRSF17-targeted immunotherapy: a high-throughput sequencing study. Front Immunol 15: 1303611, 2024. doi:10.3389/fimmu.2024.1303611. 38440734 PMC10909935

[66] Yang M, Wang P, Liu T, Zou X, Xia Y, Li C, Wang X. High throughput sequencing revealed enhanced cell cycle signaling in SLE patients. Sci Rep 13: 159, 2023. doi:10.1038/s41598-022-27310-8. 36599883 PMC9812989

[67] Xiao L, Xiao W, Lin S. Potential biomarkers for active renal involvement in systemic lupus erythematosus patients. Front Med (Lausanne) 9: 995103, 2022. doi:10.3389/fmed.2022.995103. 36530895 PMC9754094

[68] Zhang L, Chen W, Xia N, Wu D, Yu H, Zheng Y, Chen H, Fei F, Geng L, Wen X, Liu S, Wang D, Liang J, Shen W, Jin Z, Li X, Yao G, Sun L. Mesenchymal stem cells inhibit MRP-8/14 expression and neutrophil migration via TSG-6 in the treatment of lupus nephritis. Biochem Biophys Res Commun 650: 87–95, 2023. doi:10.1016/j.bbrc.2023.02.005. 36791546

[69] Aringer M, Smolen JS. Cytokine expression in lupus kidneys. Lupus 14: 13–18, 2005. doi:10.1191/0961203305lu2053oa. 15732282

[70] Lemay S, Mao C, Singh AK. Cytokine gene expression in the MRL/lpr model of lupus nephritis. Kidney Int 50: 85–93, 1996. doi:10.1038/ki.1996.290. 8807576

[71] Berthier CC, Bethunaickan R, Gonzalez-Rivera T, Nair V, Ramanujam M, Zhang W, Bottinger EP, Segerer S, Lindenmeyer M, Cohen CD, Davidson A, Kretzler M. Cross-species transcriptional network analysis defines shared inflammatory responses in murine and human lupus nephritis. J Immunol 189: 988–1001, 2012. doi:10.4049/jimmunol.1103031. 22723521 PMC3392438

[72] Shu B, Fang Y, He W, Yang J, Dai C. Identification of macrophage-related candidate genes in lupus nephritis using bioinformatics analysis. Cell Signal 46: 43–51, 2018. doi:10.1016/j.cellsig.2018.02.006. 29458096

[73] Karimifar M, Pakzad B, Karimzadeh H, Mousavi M, Kazemi M, Salehi A, Vatandoust N, Amini G, Akbari M, Salehi R. Interferon-induced protein 44-like gene promoter is differentially methylated in peripheral blood mononuclear cells of systemic lupus erythematosus patients. J Res Med Sci 24: 99, 2019. doi:10.4103/jrms.JRMS_83_19. 31850088 PMC6906918

[74] Nzeusseu Toukap A, Galant C, Theate I, Maudoux AL, Lories RJ, Houssiau FA, Lauwerys BR. Identification of distinct gene expression profiles in the synovium of patients with systemic lupus erythematosus. Arthritis Rheum 56: 1579–1588, 2007. doi:10.1002/art.22578. 17469140

[75] Gao LJ, Li JL, Yang RR, He ZM, Yan M, Cao X, Cao JM. Biological characterization and clinical value of OAS gene family in pancreatic cancer. Front Oncol 12: 884334, 2022. doi:10.3389/fonc.2022.884334. 35719943 PMC9205247

[76] Li X, Huo Y, Wang Z. Screening of potential biomarkers of system lupus erythematosus based on WGCNA and machine learning algorithms. Medicine (Baltimore) 102: e36243, 2023. doi:10.1097/MD.0000000000036243. 38013304 PMC10681579

[77] Zimmermann-Geller B, Koppert S, Fischer S, Cabrera-Fuentes HA, Lefevre S, Rickert M, Steinmeyer J, Rehart S, Umscheid T, Schonburg M, Muller-Ladner U, Preissner KT, Frommer KW, Neumann E. Influence of extracellular RNAs, released by rheumatoid arthritis synovial fibroblasts, on their adhesive and invasive properties. J Immunol 197: 2589–2597, 2016 [Erratum in J Immunol 198: 1376, 2017]. doi:10.4049/jimmunol.1501580. 27549172

[78] Lasch M, Kumaraswami K, Nasiscionyte S, Kircher S, van den Heuvel D, Meister S, Ishikawa-Ankerhold H, Deindl E. RNase A treatment interferes with leukocyte recruitment, neutrophil extracellular trap formation, and angiogenesis in ischemic muscle tissue. Front Physiol 11: 576736, 2020. doi:10.3389/fphys.2020.576736. 33240100 PMC7677187

